# Clinical and molecular characterization of a *de novo* 19p13.3 microdeletion

**DOI:** 10.1186/s13039-016-0252-x

**Published:** 2016-05-27

**Authors:** Pietro Palumbo, Orazio Palumbo, Maria Pia Leone, Raffaella Stallone, Teresa Palladino, Leopoldo Zelante, Massimo Carella

**Affiliations:** Laboratorio di Genetica Medica, IRCCS Casa Sollievo della Sofferenza, San Giovanni Rotondo (FG), Italy; Dipartimento di Scienze del suolo, della pianta e degli alimenti, Università degli Studi di Bari “Aldo Moro”, Bari, Italy

**Keywords:** 19p13.3 microdeletion, SNP-Array analysis, Intellectual disabilities

## Abstract

**Background:**

Structural rearrangements of chromosome 19p13.3 are a rare condition, and their phenotypic consequences remain not well defined, because of the variability of clinical manifestations. Increasing knowledge of new 19p13.3 microdeletion is useful to clarify the phenotypic variability observed in some patients. In a small number of recent papers, patients with intellectual disabilities, multiple congenital anomalies and microdeletion of the chromosome band 19p13.3 have been described. However, little is known about genes responsible for clinical features in patients carriers of 19p13.3 microdeletion; thus, increasing number of reported cases will be helpful to investigate the contribution of candidate genes, providing bases for future investigations.

**Case Presentation:**

Here, we report on a 10-years-old girl referred to our genetics clinic due to intellectual disability, attention deficit, behavioral and speech delay, hypotonia, facial dysmorphisms, eye anomalies and congenital malformations. Using an high resolution SNP array, we identified a *de novo* microdeletion of chromosome 19p13.3, resulting in the heterozygous loss of 27 RefSeq genes and a miRNA, partially overlapping with three others deletions already reported in literature, but extending downstream (centromeric) for additional 386 Kb. This chromosomal region includes 13 genes amongst of which we suggest for the first time the *APC2*, *PLK5* and *MBD3* genes as potential functional candidates for neurodevelopmental and behavioral phenotypes observed.

**Conclusions:**

Here we describe a patient with a 19p13.3 microdeletion that spans to the downstream chromosomal region with respect to the overlapping deletions previously reported in several other cases. The neurobehavioral features observed in our case has extended the phenotypic spectrum associated with the 19p13.3 microdeletion. New candidate genes are proposed for the neurobehavioral phenotype observed in our case.

## Background

Copy number variations (CNVs) are recognized as consisting genetic causes or susceptibility factors for neurodevelopmental disorders (NDDs), dysmorphic syndromes and multiple congenital anomalies (MCAs). CNVs can arise on all chromosomes, and their clinical manifestations depends on gene content, on the involvement of imprinted regions and on the nature of the rearrangement; furthermore, the variety of phenotypes may be due to several factors such as incomplete penetrance, variable expressivity and environmental factors. Chromosome 19 has the highest gene density, indicating its importance in several biological function [1] and, therefore, even small aberrations on this chromosome are likely to have clinical consequences. To date, with the widespread use of genome wide microarray technology, several cases of interstitial 19p13.3 microdeletion have been reported, and the increasing resolution of analysis methods allowed the improvement of genotype-phenotype correlations. These observations highlighted both common clinical features and phenotype variations among described patients, probably due to different size of the deletions and their gene content.

Moreover, some papers started to describe patients with intellectual disabilities (IDs) and MCAs associated with 19p13.3 microdeletions [2, 3], highlighting the evidence that 19p13.3 microdeletion may cause these clinical conditions. In order to deeply investigate the phenotype associated with 19p13.3 microdeletion, we describe the clinical and molecular data of a patient carrier of a 0.71 Mb *de novo* interstitial microdeletion of chromosome 19p13.3. Interestingly, the deletion identified in our patient partially overlap with those already reported in literature, and extend towards the proximal (centromeric) end for further 386 Kb. This region includes 13 genes and a miRNA, some of them seems to be functional candidates for the clinical traits observed in our patient.

## Case presentation

### Case report

**Fig. 1:**
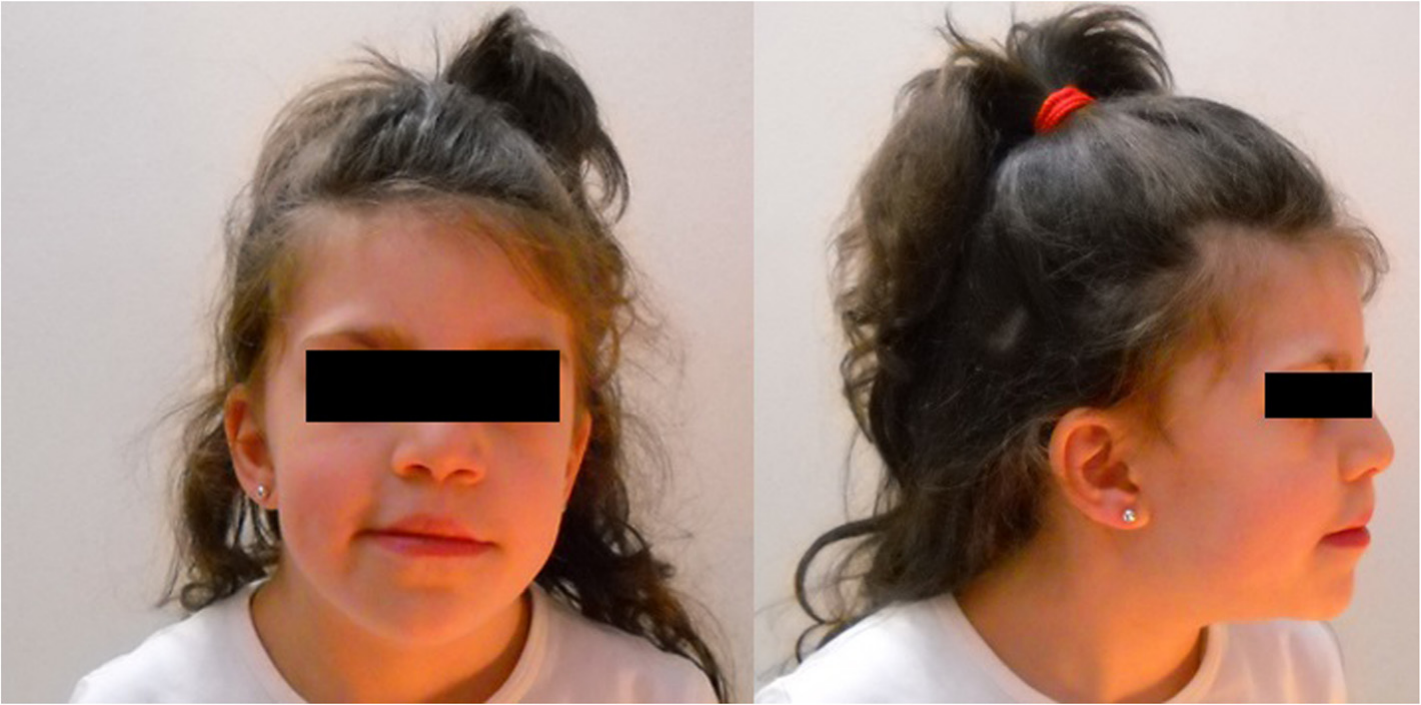
Facial phenotypes (frontal and lateral view) of the patient described in detail in the text

The patient described herein, a 10-year-old girl, is the first child of healthy non-consanguineous parents, born after an uneventful pregnancy affected by ID, attention deficit, behavioral and speech delay, hypotonia, facial dysmorphisms, eye anomalies. She also showed congenital malformations such as cerebral anomaly and forward anus. Her birth weight was 3,450 g (50th centile), length was 50,7 cm (50th centile), and head circumference was 34 cm (25-50th centile). The Apgar score was 9/9. Since the birth, the child has been subjected to a series of medical tests that revealed a complex clinical picture, such as a brain ultrasound that showed the presence of a slightly wider left ventricle. Furthermore, the presence of further forward anus relative to the sphincter floor was observed, so she was subsequently subjected to electro stimulation and submitted to surgery for the removal of vegetation skin. At 30 days visit, a cranial ultrasound showed a slightly dilated left ventricle, while there were not cardiologic alterations or skeletal anomalies. A brain MRI (Magnetic Resonance Imaging) confirm the presence of a wider left ventricle. Standard karyotype was normal, as well as X-Fragile test. The neuropsychiatric evaluation performed at the age of 4.7 years showed an I.Q. of 89 (Stanfod Binet scale) and a mental age of 3 years and 10 months. At last clinical evaluation, performed when she was 10 years old, the patient showed facial dysmorphisms such as prominent mandible and enlarged nasal root (Fig. 1), in association with mild hypotonia, a hint of curvature of the trunk; a comprehensive neuropsychiatric evaluation has also been performed. The girl showed mild ID, with a greater operating capacity of verbal thought than visual-motor thought. If properly stimulated, the patient offers the best overall performance; she showed also graphomotor delay, handling and grip difficulties. Regarding the affective and relational behavior, the child was introverted and showed attention deficit. About the communication and language area, the child showed speech delay (dyslexia). Finally, audiological tests showed normal hearing, and eye exams showed exotropia.

## Results

**Fig. 2:**
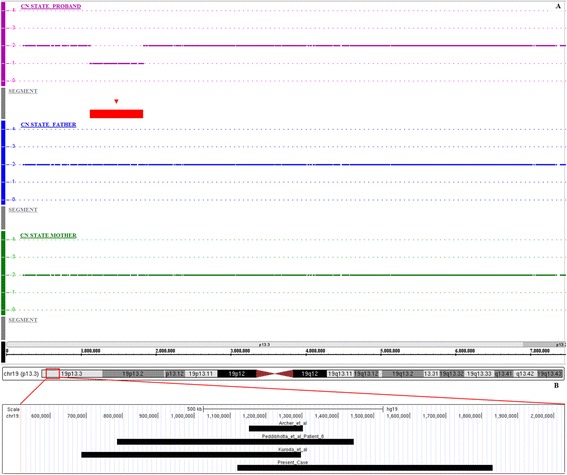
**a** Microarray-based copy number analysis performed with the Affymetrix Genome Wide Human SNP 6.0 array and visualized using the Affymetrix Genotyping Console Browser. Copy number state of each probe is drawn along chromosome 19 from 0 to 7,000,000 bp. The upper panel represents the copy number state of the proband, the middle panel the father and the lower panel the mother. Values of Y-axis indicate the inferred copy number according to probe intensities. Red bar is the deleted region identified in the patient. **b** Localization of overlapping deletions identified in our patient and in patients already described in literature

SNP-array analysis of the patient revealed a heterozygous deletion involving chromosome 19p13.3. The deleted region was 710 Kb in size and covered by 159 SNP array probes. The proximal breakpoint (telomeric) was located between the last present probe CN_780351 (1,118,914 bp) and the first deleted probe SNP_A-8630732 (1,120,329 bp), while the distal breakpoint (centromeric) was located between the last deleted probe CN_782662 (1,829,934 bp) and the first present probe SNP_A-1937744 (1,837,061 bp) (USCS Genome Browser build February 2009 hg19). Consequently, the maximum size of the deletion is 718 Kb while the minimum size is 710 Kb. The subsequent microarray analysis of the patient’s parents, by using the same platform, revealed normal chromosomes 19 in both of them, indicating a *de novo* deletion in the child (Fig. 2a). No additional rare CNVs were detected in her genome.

According to the International System for Human Cytogenetic Nomenclature (ISCN 2013), the molecular karyotype of the patient was arr[hg19]19p13.3(1,118,914x2,1,120,328-1,829,934x1,1,837,061x2)dn. This rearrangement was not reported as a benign CNV in the Database of Genomic Variants (http://projects.tcag.ca/variation/) and includes 27 genes and a miRNA. The deletion in the patient was confirmed by a second experiment using a lower resolution array (Cytoscan 750 K Array, Affymetrix, Santa Clara, CA). The deleted region was 645 Kb in size (slightly smaller than the one identified by using the SNP 6.0 array, due to the lower resolution of the assay) and covered by 125 SNP array probes. The first deleted probe was at 1,170,883 bp (C-3XHQX), while the last deleted probe was at 1,816,237 bp (C-6ADPS) (USCS Genome Browser build February 2009 hg19).

## Discussion

Interstitial microdeletions of chromosome 19p13.3 are rarely described. We report on a 10-year-old girl affected by ID, language and behavioral delay, attention deficit, facial dysmorphisms and eye anomalies in addition to hypotonia and MCAs carriers of a *de novo* 19p13.3 interstitial microdeletion, 0.71 Mb in size, identified by SNP-array analysis. To date, only three other cases with overlapping rearrangement, similar in size and chromosomal position, have been reported in medical literature. Clinical manifestation and molecular data of our patient and previous cases with 19p13.3 microdeletion are listed in Table 1 and showed in Fig. 2b.

**Table 1:** Clinical manifestation and molecular data of our patient and previous cases with 19p13.3 microdeletion

	Archer et al.	Peddibhotla et al., Patient 8	Kuroda et al.	DECIPHER 253691	Present case
Age at diagnosis (years)	16	3	12	Unknow	10
Sex	Male	Female	Female	Male	Female
Coordinates of 19p13.3 microdeletion (hg19)	1,152,413–1,302,309 bp	785,691–1,444,289 bp	686,663–1,297,499 bp	1,272,198–2,138,731Bp	1,120,328–1,829,934 bp
Inheritance	*de novo*	N.R.	*de novo*	unknown	*de novo*
Neurocognitive					
DD/ID Speech delay	+ -	+ +	+ -	+ -	+ +
Neurologic				N.R.	
Seizures Hypotonia Motor delay Dyspraxia EEG anomalies	+ + - + +	- + + - +	+ + - - -		- + + - -
Neuropsychiatric				N.R.	
Behavioral delay Attention deficit	- -	- -	- -		+ +
Hearing impairement	+	-	+	N.R.	-
Eye anomalies Exotropia Myopia Strabismus	- - -	- - +	- + +	N.R.	+ - -
Facial dysmorphisms	+	+	+	+	+
Congenital anomalies				N.R.	
Cleft palate Renal agenesis Atrial septal defect Ventricular septal defect Umbilical hernia Hiatal hernia Inguinal Hernia Scoliosis Further forward anus	+ - - + + - - - -	- + + - + + - - -	- - - - + - + + -		- - - - - - - + +
Cerebral anomalies				N.R.	
Ventricular anomalies	+	+	-		+

*DD* developmental delay, *ID* intellectual disability, *bp* base pairs, + feature present, − feature absent *N.R.* not reported

In 2005, Archer et al. [4] described a patient, carrier of a 0.15 Mb microdeletion on chromosome 19p13.3, affected by learning difficulties/ID, dyspraxia, hypotonia, cleft palate, unusual facial appearance, sensorineural deafness, congenital heart defect, a prominent lateral ventricle, seizure and immune deficiencies. In this case, the authors suggested the haploinsufficiency of *EFNA2* as possible cause for the phenotypes observed. This patient share some clinical features with our (ID, hypotonia, and cerebral anomalies).

Later, Peddibhota and colleagues [2] described eight cases of 19p13.3 microdeletion, one of which partially overlapped with our case. The clinical manifestations of this patient, a 3-years-old girl, was characterized by developmental delay (DD), moderate ventriculomegaly, sub-optimal weight gain, joint laxity, right-sided ptosis, strabismus, hiatal hernia, small umbilical hernia, hydrocephalus, broad forehead, widely spaced deeply set eyes, midface retrusion, asymmetric ears, pointy chin, hypotonia, delayed speech and motor development. In this case, none of the candidate genes suggested by the authors as responsible for the onset of the phenotype (*ELANE, MED16* and *ARID3A*) are included in the chromosomal region deleted in our patient, even if some clinical features are common, such as DD/ID, hypotonia, delayed speech and motor development, ventricular anomalies.

More recently, Kuroda et al. [5] described a 12 years old girl carriers of a 19p13.3 microdeletion, 0.61 Mb in size detected by array Comparative Genomic Hybridization (aCGH), affected by Peutz Jeghers syndrome (PJS). In addition, the patient showed ID (I.Q. 46), hypotonia, nail pigmentation, umbilical hernia, bilateral inguinal hernias, scoliosis, myopia, conductive deafness secondary to middle ear disease, and distinctive facial features (long face, pointed chin, broad eyebroes, thin lip vermillion, smooth philtrum, long hanging columella and malformed ears). The authors identified *STK11* gene as responsible for PJS features, and suggested two other genes, *ARID3A* and *PTBP1*, as responsible for the ID. The patient described by Kuroda and colleagues share some clinical features with our patient, such as hypotonia, ID and facial dysmorphisms, but candidate genes are not shared between them, with the exception of *STK11.*

Finally, in DECIPHER we found patient 253691, affected by abnormality of the face and intellectual disabilities, thus similar to the phenotypes observed in our patient. Even in this case the deleted region did not encompass *STK11*, but other genes including *APC2*, *PLK5* and *MBD3*. Respect to the three cases reported and discussed above, our patient shows behavioral delay and marked attention deficit which may be added into the clinical spectrum associated with the 19p13.3 microdeletion. Obviously more patient are needed to delineate the full spectrum of phenotypes due to the 19p13.3 microdeletion.

From a molecular point of view, it is interestingly to note that while previous reports describe patients carriers of a 19p13.3 microdeletion involving a chromosomal region partially overlapping with the microdeletion identified by us*,* our case is the first one to date reported in which the deletion span downstream respect other reported deletions, involving genes which are new candidates for the clinical manifestations. In detail, the 19p13.3 microdeletion described here encompass 27 genes (*SBNO2, STK11, C19orf26, ATP5D, MIDN, CIRBP-AS1, CIRBP, C19orf24, EFNA2, MUM1, NDUFS7, GAMT, DAZAP1, RPS15, APC2, C19orf25, PCSK4, REEP6, ADAMTSL5, PLK5, MEX3D, MBD3, UQCR11, TCF3, ONECUT3, ATP8B3, REXO1*) and a miRNA (*MIR1909*), partially overlap with those previously reported (from *SBNO2* to *RPS15* genes) and extends towards the proximal (centromeric) ends for additional 386 Kb (from 1,444,289 to 1,829,934 bp). This additional chromosomal region includes 13 genes (from *APC2* to *REX01*), some of which could be involved in the observed clinical phenotype.

Between the genes located inside the deleted 19p13.3 region shared by all reported patients, we suggest as candidates for the observed phenotypes *STK11, MIDN* and *EFNA2A.*

*STK11* encodes a member of the serine/threonine kinase family, regulates cell polarity and functions as a tumor suppressor, controls the activity of AMP-activated protein kinase (AMPK) family members, thereby playing a role in a wide range of biological processes such as cell metabolism, cell polarity, apoptosis, cortical neurons polarization, axon initiation and specification, DNA damage response [6]. Diseases associated with *STK11* include testicular tumor, somatic, and intussusception [6]. Because of its involvement in cortical neuronal processes, we can suppose that haploinsufficiency of *STK11* could be associated to the neurobehavioral phenotype documented in our patient; furthermore, it could be involved in the development of the skin vegetation observed in our patient. Also, deletions and point mutations of *STK11* are the major causes of PJS [7]. Although our patient did not show any features of this condition at the time of clinical evaluation, we cannot exclude that she will manifest later the clinical traits of this syndrome. Thus, we suggest a clinically surveillance for the PJS.

*MIDN* (midnolin) is a gene potentially involved in regulation of genes related to neurogenesis in the nucleolus [8], was already reported in a study aimed to identify novel autism related genes [6], thus it could contribute to the onset of DD/ID.

*EFNA2* (ephrin-A2) encodes for a member of the ephrin family. The EPH and EPH-related receptors comprise the largest subfamily of receptor protein-tyrosine kinases which have been implicated in mediating developmental events, particularly in the nervous system [9]. Since the functional evidences and its expression profile, in agreement with Archer and colleagues [4], we consider this gene as a possible candidate for the ID described in our patient. In addition, functional studies demonstrated the high level of expression of this gene also during eye and retina development [10], therefore it is reasonable to assume that *EFNA2* can be involved in the onset of eye anomalies observed. Even if some authors suggested that the haploinsufficiency of these two genes (*EFNA2* and *MIDN*) did not impact on the phenotype [3], their involvement cannot be excluded, also because they have been already described as associated to clinical conditions [11].

Between the 13 genes located inside the chromosomal region deleted only in our patient (from 1,444,289 to 1,829,934 bp), some deserve special attention. According to their biological functions and their expression profiles, *APC2*, *PLK5* and *MBD3* may have contributed to the neurodevelopmental and behavioral phenotypes observed in our patient.

The first one, *APC2* (adenomatosis polyposis coli 2), also called *APCL,* is primary expressed in central nervous system, preferentially in post-mitotic neurons throughout development, and it is involved in brain development through the regulation of neuronal migration and axon guidance [12]. APC2 protein could bind to beta-catenin and deplete the intracellular beta-catenin pool, suggesting that APC2 is involved in the regulation of neuronal function in association to beta-catenin [13]. Furthermore, it was already associated to human genetic neurological disorder [14] and Apc2-deficient mice (Apc2^−/−^) exhibited impaired learning and memory abilities along with abnormal head shape [15]. Thus, since these functional and clinical evidences, *APC2* seems to be a good functional candidate gene for the onset of neurodevelopmental phenotype observed in our patient.

*PLK5* (Polo-like kinases 5) is a member of a protein family characterized by the presence of a specific domain, known as the polo box (PDB), involved in protein-protein interactions. *Plk1 to Plk4* are involved in centrosome biology as well as the regulation of mitosis, cytokinesis and cell cycle checkpoints in response to genotoxic stress [16]. On the contrary, *Plk5* does not seems involved in these mechanisms, because is mostly expressed in the brain of both mice and humans, and it modulates the formation of neuritic processes upon stimulation of the brain-derived neurotrophic factor (BDNF)/nerve growth factor (NGF)-Ras pathway in neurons [17]. Due to its involvement in neuronal processes, its contribute to the clinical manifestation in our patient cannot be excluded. Finally, a gene that may be involved in the onset of cognitive and behavioral phenotypes observed in our patient is *MBD3* (methyl-CpG binding domain protein 3). This gene belongs to a family of nuclear proteins which are characterized by the presence of a methyl-CpG binding domain (MBD). The encoded protein is a subunit of the NuRD, a multisubunit complex containing nucleosome remodeling and histone deacetylase activities. Unlike the other family members, the encoded protein is not capable of binding to methylated DNA. The protein mediates the association of metastasis-associated protein 2 with the core histone deacetylase complex. A component of the same gene family, *MBD5*, has been associated to neurobehavioral abnormalities by Camarena and colleagues [18], which reported the evidence that disruption of Mbd5 in mice causes neuronal functional deficits and neurobehavioral abnormalities. Even if *MBD3* has never been associated to neurobehavioral disorders, the evidence that another member of the same gene family cause a similar clinical phenotype may be suggestive of its contribution in the onset of the observed traits.

## Conclusion

In conclusion, we hypothesize that 19p13.3 microdeletions are responsible of a wide spectrum of phenotype including ID, MCAs and dysmorphic features. Our patient has showed a microdeletion that overlaps with the deletion region reported in several previous cases and extends 386 Kb downstream. Phenotype comparison of these cases suggests that deletion of the *APC2*, *PLK5* and *MBD3* genes may have contributed to behavioral delay and marked attention deficit observed in our case. Obviously more cases, carriers of deletions encompassing candidate genes proposed in this paper, are needed to corroborate our hypothesis and to confirm their involvement in the observed traits.

## Methods

### SNP array analysis

Blood was obtained from the proband and his parents after signed informed consent. The study was designed to perform parental and proband samples concurrently on three individual chips. Genomic DNA was isolated from peripheral blood lymphocytes of the patient and the parents by using BioRobot EZ1 (Quiagen, Solna, Sweden). DNA concentration and purity were determined with a ND-1000 Spectrophotometer (NanoDrop Technologies, Berlin, Germany) while SNP-array analysis was performed by using Genome Wide Human SNP 6.0 Array (Affymetrix, Santa Clara, CA). This array has, on average, an inter-marker distance of 700 bp and makes possible an excellent assessment of possible breakpoints of deletions to assess copy gains or losses. The SNP 6.0 assay was performed according to the manufacturer's protocol, starting with 500 ng of DNA. Briefly: total genomic DNA was digested with restriction enzymes (NspI and StyI), ligated to appropriate adapters for the enzymes, and subjected to PCR amplification using a single primer. After digestion with DNase I, the PCR products were labeled with a biotinylated nucleotide analog using terminal deoxynucleotidyl transferase and hybridized to the microarray. Hybridization was carried out in the Affymetrix Hybridization Owen 450 while subsequent washing and staining steps were performed using the Fluidic Station 450 and finally the array was scanned with the GeneChip Scanner 3000 7G using Command Console Software (Affymetrix, Santa Clara, CA, USA). Analysis was performed using Genotyping Console Software 4.1 (Affymetrix, Santa Clara, CA, USA) and Birdseed v2-algorithm. Samples were normalized against 270 International HapMap samples while an additional 80 in-house control samples, which were hybridized in our facility, were used to decrease technical variation. For the copy number analysis, we used regional GC correction and required 50 markers to be found within the changed region and the size of the region to be at least 100 kb. The amplified and/or deleted regions were detected using a standard Hidden Markov Model (HMM) method. Genotyping Console Browser (Affymetrix, Santa Clara, CA, USA) was used to illustrate changes detected while the University of California Santa Cruz (UCSC) Genome Browser (http://genome.ucsc.edu/cgi-bin/hgGateway), assembly GRCh37, was used to map the genomic coordinates and find the genes within the copy number altered region.

## Abbreviations

aCGH, array comparative genomic hybridization; CNVs, copy number variations; DD, developmental delay; IDs, intellectual disabilities; MCAs, multiple congenital anomalies; MRI, magnetic resonance imaging; NDDs, neurodevelopmental disorders; PJS, Peutz Jeghers syndrome.
